# Epidemiological and evolutionary dynamics of influenza B virus in coastal Kenya as revealed by genomic analysis of strains sampled over a single season

**DOI:** 10.1093/ve/veaa045

**Published:** 2020-08-16

**Authors:** Festus M Nyasimi, David Collins Owuor, Joyce M Ngoi, Alexander G Mwihuri, Grieven P Otieno, James R Otieno, George Githinji, Joyce U Nyiro, David James Nokes, Charles N Agoti

**Affiliations:** Epidemiology and Demography Department, Kenya Medical Research Institute (KEMRI) – Wellcome Trust Research Programme, P.O. Box 230, Kilifi-80108, Kenya; Department of Public Health, School of Health and Human Sciences, Pwani University, P.O. Box 195, Kilifi-80108, Kenya; Epidemiology and Demography Department, Kenya Medical Research Institute (KEMRI) – Wellcome Trust Research Programme, P.O. Box 230, Kilifi-80108, Kenya; Epidemiology and Demography Department, Kenya Medical Research Institute (KEMRI) – Wellcome Trust Research Programme, P.O. Box 230, Kilifi-80108, Kenya; Epidemiology and Demography Department, Kenya Medical Research Institute (KEMRI) – Wellcome Trust Research Programme, P.O. Box 230, Kilifi-80108, Kenya; Epidemiology and Demography Department, Kenya Medical Research Institute (KEMRI) – Wellcome Trust Research Programme, P.O. Box 230, Kilifi-80108, Kenya; Epidemiology and Demography Department, Kenya Medical Research Institute (KEMRI) – Wellcome Trust Research Programme, P.O. Box 230, Kilifi-80108, Kenya; Epidemiology and Demography Department, Kenya Medical Research Institute (KEMRI) – Wellcome Trust Research Programme, P.O. Box 230, Kilifi-80108, Kenya; Epidemiology and Demography Department, Kenya Medical Research Institute (KEMRI) – Wellcome Trust Research Programme, P.O. Box 230, Kilifi-80108, Kenya; Epidemiology and Demography Department, Kenya Medical Research Institute (KEMRI) – Wellcome Trust Research Programme, P.O. Box 230, Kilifi-80108, Kenya; Department of Public Health, School of Health and Human Sciences, Pwani University, P.O. Box 195, Kilifi-80108, Kenya; School of Life Sciences and Zeeman Institute for Systems Biology and Infectious Disease Epidemiology Research (SBIDER), University of Warwick, Coventry, CV4, 7AL, UK; Epidemiology and Demography Department, Kenya Medical Research Institute (KEMRI) – Wellcome Trust Research Programme, P.O. Box 230, Kilifi-80108, Kenya; Department of Public Health, School of Health and Human Sciences, Pwani University, P.O. Box 195, Kilifi-80108, Kenya

**Keywords:** Kenya, influenza B, transmission, genomics, evolution, reassortment

## Abstract

The genomic epidemiology of influenza B virus (IBV) remains understudied in Africa despite significance to design of effective local and global control strategies. We undertook surveillance throughout 2016 in coastal Kenya, recruiting individuals presenting with acute respiratory illness at nine outpatient health facilities (any age) or admitted to the Kilifi County Hospital (<5 years old). Whole genomes were sequenced for a selected 111 positives; 94 (84.7%) of B/Victoria lineage and 17 (15.3%) of B/Yamagata lineage. Inter-lineage reassortment was detected in ten viruses; nine with B/Yamagata backbone but B/Victoria NA and NP segments and one with a B/Victoria backbone but B/Yamagata PB2, PB1, PA, and MP segments. Five phylogenomic clusters were identified among the sequenced viruses; (i), pure B/Victoria clade 1A (*n* = 93, 83.8%), (ii), reassortant B/Victoria clade 1A (*n* = 1, 0.9%), (iii), pure B/Yamagata clade 2 (*n* = 2, 1.8%), (iv), pure B/Yamagata clade 3 (*n* = 6, 5.4%), and (v), reassortant B/Yamagata clade 3 (*n* = 9, 8.1%). Using divergence dates and clustering patterns in the presence of global background sequences, we counted up to twenty-nine independent IBV strain introductions into the study area (∼900 km^2^) in 2016. Local viruses, including the reassortant B/Yamagata strains, clustered closely with viruses from neighbouring Tanzania and Uganda. Our study demonstrated that genomic analysis provides a clearer picture of locally circulating IBV diversity. The high number of IBV introductions highlights the challenge in controlling local influenza epidemics by targeted approaches, for example, sub-population vaccination or patient quarantine. The finding of divergent IBV strains co-circulating within a single season emphasises why broad immunity vaccines are the most ideal for influenza control in Kenya.

## 1. Introduction

Human influenza B virus (IBV) is responsible for about 30 percent of the influenza virus morbidity and mortality during seasonal influenza epidemics (Paul Glezen et al. 2013; Seleka et al. 2017; Caini et al. 2018a). Influenza disease burden is notably highest in low-income countries, majority of which are located in the tropics (Caini et al. 2018a; Sambala et al. 2018). Influenza virus activity in these regions tends to continue throughout the year characterised by a single or multiple epidemic peaks (Hirve and World Health Organization 2015; Caini et al. 2018a; El Guerche-Seblain et al. 2019; Emukule et al. 2019). However, understanding of IBV evolutionary dynamics and molecular epidemiology in these regions, especially in sub-Saharan Africa, remains limited with few genomes available to-date for detailed investigations (Langat et al. 2017).

Currently, there are two major IBV lineages co-circulating, B/Yamagata and B/Victoria, which diverged in the early 1970s (Kanegae et al. 1990; Rota et al. 1990; McCullers, Saito, and Iverson 2004). Differences between the two lineages are seen in their transmissibility and genetic and antigenic dynamics (Langat et al. 2017). For instance, B/Victoria lineage viruses have been shown to infect children more commonly than B/Yamagata lineage viruses (Tan et al. 2013; Vijaykrishna et al. 2015; Xu et al. 2015). Furthermore, the B/Victoria lineage viruses display a clear antigenic drift of a single clade in successive years with strong seasonal fluctuations in their incidence, whereas the B/Yamagata lineage viruses exhibit continuous co-circulation of multiple genetic clades that alternate in their dominance over years (Langat et al. 2017).

Human influenza vaccines that include one or both the two known IBV lineages are currently available. Because of the continuous antigenic evolution inherent in influenza viruses, these vaccines are periodically updated in their antigenic composition (Bedford et al. 2014). Understanding of the prevailing global and local influenza molecular epidemiology is a key consideration during selection of influenza strains to include in vaccines for upcoming seasons and for understanding observed vaccine effectiveness (Rajao and Perez 2018). However, such information is frequently unavailable for majority of developing countries (Caini et al. 2015, 2018a). Furthermore, most developing countries lack a national influenza vaccination policy (Dawa et al. 2019).

Kenya is a lower middle-income country in East Africa and is currently engaged in the process of formulating its national influenza vaccination policy (Dawa et al. 2019). The country lies on the equator with a climate that varies regionally, mostly between tropical to sub-tropical. An influenza surveillance study conducted between 2012 and 2016 across eleven sites in Kenya found that approximately 31% of medically attended influenza cases were of IBV type (Emukule et al. 2019). IBV prevalence among influenza positives fluctuated from year-to-year, with 2016 recording the highest proportion (61%) over the 5-year surveillance period. Furthermore, it was observed that the two IBV lineages alternated in predominance; B/Victoria lineage predominated in the years 2012 and 2016, whereas B/Yamagata lineage predominated in the years 2013, 2014, and 2015 (Emukule et al. 2019).

Influenza genomic analysis is now recognised as instrumental in providing a detailed information on the mutations that could facilitate antigenic escape, antiviral resistance, enhanced virulence and can uncover the transmission history and pathways of locally and globally circulating viruses (Hirve and World Health Organization 2015; Goldstein et al. 2018). Unlike influenza A, no study to-date has examined the genomic epidemiology of IBV in Kenya or elsewhere in Africa to characterise the local phylodynamics and phylogeography in comparison with known global patterns. The extent of inter-connectedness of IBV epidemics that occur locally to those happening regionally and globally is yet to be examined. In this study, we present detailed genomic analysis of the circulating IBV strains over a single year in a rural coastal area of Kenya, their spread, evolutionary dynamics, and global context.

## 2. Materials and methods

### 2.1 Study area and population

All samples analysed here were collected in health facilities within the Kilifi Health and Demographic Surveillance System (KHDSS) area (Scott et al. 2012), located at the Indian ocean coastal region of Kenya. The study period was over a 1 year period (January–December 2016; Nyiro et al. 2018). The KHDSS area spans ∼900 km^2^ and is resident to ∼280,000 people as at of 2016. The area has a tropical climate with two rainy seasons; the main rains that usually peak in April and the short rains that usually peak in November (Nokes et al. 2009). Study participants were both resident (majority) and non-resident individuals of any age presenting with acute respiratory illness (ARI) to nine primary outpatient health facilities within the KHDSS area (Nyiro et al. 2018) or inpatients under 5 years of age admitted with syndromic severe or very severe pneumonia to the Kilifi County Hospital (KCH; Nokes et al. 2009).

### 2.2 Study design

**Figure 1.**
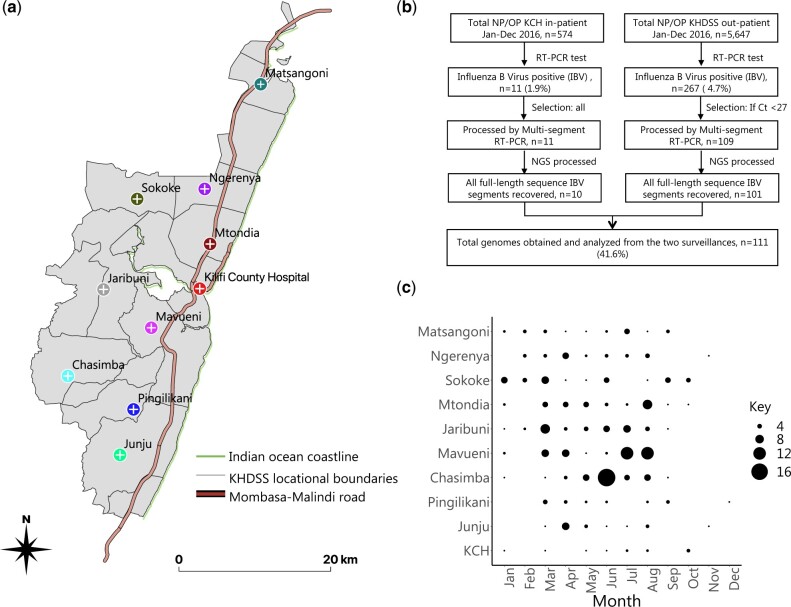
Study location, sample laboratory processing, and IBV detection in the enrolled health facilities. (a) A map of the Kilifi Health and Demographic Surveillance System (KHDSS) area showing the spatial distribution of the enrolled KHDSS health facilities. (b) A sample flow gram showing the number of samples and genomes obtained from the Kilifi County Hospital (KCH) and KHDSS outpatient facilities surveillances. (c) A bubble plot showing the number of IBV positives by month and health facility across 2016. The size of the circle is proportional to the number of samples (smallest represent one and the largest represent seventeen samples).

The description of the study design, including selection criteria and case definitions, at KCH and at the included KHDSS outpatient facilities can be found in our previous reports (Nokes et al. 2009; Nyiro et al. 2018). KCH is main referral hospital in Kilifi County providing inpatient care and is located in Kilifi town, the county headquarters. All children meeting the inclusion criteria are eligible for enrolment (around 75% are approached, consented, and a sample collected). The outpatient facilities included in the study were Chasimba, Sokoke, Matsangoni, Ngerenya, Mavueni, Mtondia, Junju, Jaribuni, and Pingilikani, Fig. 1a. The study aimed to collect a sample from the first fifteen eligible individuals identified in each clinic per week (7,020 samples in total). Roughly equal numbers of samples were collected from each outpatient facility throughout the year (Nyiro et al. 2018), with approximately similar numbers collected per month except for December which was affected by health workers strike (Ong’ayo et al. 2019). Samples from KCH represented a full-year sampling fulfilling the eligibility criteria.

### 2.3 Ethical approval

The KEMRI Scientific and Ethics Review Unit (SERU) granted ethical clearance for the study protocol and procedures. Study participants or their parents/caregivers (if aged < 18 years) provided a written informed consent to participate in this study before sample collection.

### 2.4 Sample handling and IBV detection

Nasopharyngeal (NP) swabs were collected at the outpatient facilities, whereas naso-oropharyngeal (NP/OP) swabs were collected at KCH. The swabs were immediately put into viral transport medium and transferred to a cool box with ‘ice’ blocks before transportation to the Kenya Medical Research Institute (KEMRI)—Wellcome Trust Research Programme laboratories (KWTRP) for long-term storage at −80 °C. All samples were screened for fifteen different viruses using real-time reverse transcription polymerase chain reaction (RT-PCR) diagnostic assay, as previously described (Hammitt et al. 2011; Onyango et al. 2012). IBV primers and probe targeted a conserved region of the non-structural (NS) segment of IBV genome (Gunson and Carman 2011). IBV positive samples with a considerably high viral load as defined an RT-PCR cycle threshold (*Ct*) value of <27.0, were selected for the KHDSS outpatient facilities arm, but all IBV positive samples regardless of *Ct* value from KCH inpatients arm, for whole-genome sequencing, Fig. 1b.

### 2.5 Nucleic acid extraction and multi-segment RT-PCR

Nucleic acid was extracted from IBV positive samples using the QIAamp Viral RNA Mini Kit (Qiagen, Hilden, Germany). Complete IBV segments were amplified using the Universal IBV-GA2 primer set (Zhou et al. 2014) in a multi-segment RT-PCR using SuperScript III One-Step Kit with Platinum Taq DNA Polymerase High Fidelity (Invitrogen, Carlsbard, CA, USA). The universal primers amplify all eight IBV genome segments in a single PCR tube (Zhou et al. 2014). Successful amplification was confirmed by running the PCR products and controls on a 2 per cent agarose gel and visualising on a UV trans-illuminator after staining with RedSafe Nucleic Acid Staining solution (iNtRON Biotechnology Inc., Seoul, South Korea).

### 2.6 Next generation sequencing

Amplicons were purified with 1×AMPure XP beads (Beckman Coulter Inc., Brea, CA, USA), quantified with Quant-iT dsDNA high-sensitivity assay (Invitrogen), and normalised to 0.2 ng/µl. Indexed paired-end libraries were generated from 2.5 µl of 0.2 ng/µl amplicon pool using Nextera XT sample Preparation Kit (Illumina, San Diego, CA, USA) following the manufacturer’s protocol. Amplified libraries were purified using 0.8 × AMPure XP beads, quantified using the Quant-iT dsDNA high-sensitivity assay, and evaluated for fragment size in the Agilent 2100 BioAnalyzer System using the Agilent High Sensitivity DNA Kit (Agilent Technologies, Santa Clara, CA, USA). Libraries were diluted to 2 nM, pooled, and denatured then diluted to 12.5 pM. Sequencing was performed on the Illumina MiSeq using MiSeq v250 Cycle Kit with 5 per cent PhiX (Illumina) spike-in. Sequence assembly was performed using the Iterative Refinement Meta-Assembler (IRMA) default settings: median read Q-score filter of 30, the minimum read length of 125, the frequency threshold for insertion and deletion refinement of 0.25 and 0.60, respectively; Smith–Waterman mismatch penalty of 5; and gap opening penalty of 10 (Shepard et al. 2016).

### 2.7 Comparison dataset

Two datasets compiled from the Global Initiative on Sharing All Influenza Data (GISAID) were prepared for comparison with the newly sequenced Kilifi IBV strains. The first dataset comprised reference sequences of B/Victoria and B/Yamagata lineages, and clades within these lineages collected between 1987 and 2020, and IBV strains that were included in trivalent and quadrivalent influenza vaccines that were recommended during the 2015–16 northern hemisphere influenza season (https://www.who.int/influenza/vaccines/virus/recommendations/2015_16_north/en/). This dataset (*n* = 54) was used to assign lineage and clades to the Kilifi IBV strains and in segment-by-segment evolutionary analyses. The second dataset was a 1,207 sample of IBV whole genomes deposited in GISAID database for samples collected between January 2014 and December 2016 across all continents. Details on how these were selected are provided in Supplementary Table S1. This dataset was used to investigate the global phylogenetic context of the Kilifi IBV strains.

**Table 1.** Clinical and demographic characteristics of patients who were IBV positive and a comparison of those sequenced versus those not sequenced.

Characteristic	All positives (*n* = 278)	Sequenced (*n* = 111)	Not sequenced (*n* = 167)	*P* value
Health facility				
Inpatient (KCH)	11 (4.0%)	10 (9.0%)	1 (1.0%)	0.001
Outpatient (KHDSS)	267 (96.0%)	101 (91.0%)	166 (99.0%)	
Age (years)				
Mean (SD)^€^	9.6 (13.3)	9.7 (15.2)	9.6 (11.9)	0.938
Median (IQR)	6 (2–12)	6 (2–11)	5 (2–13)	0.924
Age class (years)				
0–4	120 (43.2%)	49 (44.2%)	71 (42.5%)	0.203
5–14	109 (39.2%)	47 (42.3%)	62 (37.1%)	
15–34	32 (11.5%)	9 (8.1%)	23 (13.8%)	
35–64	13 (4.7%)	3 (2.7%)	10 (6.0%)	
≥65	4 (1.4%)	3 (2.7%)	1 (0.6%)	
Gender				
Female	159 (57.2%)	65 (58.6%)	94 (56.3%)	0.708
Male	119 (42.8%)	46 (41.4%)	73 (43.7%)	
Clinical symptoms				
Fever	220 (79.1%)	89 (80.2%)	131 (78.4%)	0.727
Cough^a^	260 (97.4%)	98 (97.0%)	162 (97.6%)	0.781
Nasal discharge^a^	204 (76.4%)	79 (78.2%)	125 (75.3%)	0.586
Breathing difficulty	28 (10.1%)	15 (13.5%)	13 (7.8%)	0.120
Viral load (*Ct* value)				
Mean (SD)	27.7 (3.2)	25.0 (2.3)	29.5 (2.4)	<0.001
Median (IQR)	27.6 (25.7–29.6)	25.2 (23.5–26.5)	29.2 (28.0–31.0)	<0.001

^a^ *n* = 267, 101, and 166 for the categories; all positives, those sequenced, and those not sequenced, respectively. Symptoms data were unavailable for inpatients. SD, Standard of Deviation; IQR, Interquartile range.

### 2.8 Phylogenetic analysis

The assembled and segment assorted Kilifi nucleotide (nt) sequences were aligned together with the global references using MAFFT v7.245 (Katoh and Standley 2013) and visualised using Aliview v1.25 (Larsson 2014). For each of segment dataset, maximum likelihood (ML) phylogenetic trees were inferred using RaxML v.8.2.12 (Stamatakis 2014), based on the best-fit models of nt substitution determined by IQ-TREE v1.5.5 (Nguyen et al. 2015). The individual segment sequences were concatenated to give the full-length genome sequences using SequenceMatrix (Vaidya, Lohman, and Meier 2011). For all ML trees, the clustering reliability was evaluated by bootstrap resampling 1,000 replicates. The tree topologies across segments were examined to detect reassortment events, using FigTree v1.4.4 (http://tree.bio.ed.ac.uk/software/figtree/). Reassortant IBV strains were confirmed computationally using the Graph-incompatibility-based Assortment Finder (GiRaF) tool (Nagarajan and Kingsford 2011).

### 2.9 Evolutionary analysis

This was undertaken for the individual segments and the concatenated genomes. The linearity in nt sequence divergence with sampling time for datasets used in inferring the time to the Most Recent Common Ancestor (tMRCA) and substitution rates were initially assessed using TempEst v1.5.3 programme (Rambaut et al. 2016). Viruses suspected to be reassortants were analysed separately. Nt substitution rates and the tMCRA were estimated using the Bayesian approach implemented in BEAST v1.10.4 program (Suchard et al. 2018). The log files from the analysis were examined using Tracer v1.7.1 program (http://tree.bio.ed.ac.uk/software/tracer/) and run convergence (defined as estimated sample size of > 100 for all sampled parameters) was confirmed before extracting the relevant parameter estimates. All BEAST runs were set to at least 10 million steps with sampling after every 2,500 steps. Additional and longer runs were considered if the initial analysis did not show convergence. Maximum clade credibility (MCC) trees were summarised from the tree log file using TreeAnnotator v1.10.4 with a 10 percent burn-in. The MCC trees were visualised using FigTree v1.4.4 (Suchard et al. 2018).

### 2.10 Genetic diversity and transmission analysis

We categorised the diversity observed in the Kilifi sequenced viruses using five measures that reflected the closeness of the viruses in their underlying transmission history and genetic diversity. These levels were defined on the basis of the observed phylogenetic clustering with reference sequences, the concatenated segments phylogeny (that identified reassortants), pairwise nt distances, and the estimated time of divergence at the branch nodes. The defined categories (the first two consistent with what has been described in literatures) are summarised below:

- *Lineage*: Based on phylogenetic clustering of HA segment sequences with B/Yamagata and B/Victoria lineage reference sequences (Arvia et al. 2014).
- *Clade*: Based on HA phylogenetic clustering with reference sequences of known clades within B/Yamagata (Clades 1–3) and B/Victoria (Clades 1–6) lineages (Arvia et al. 2014; Tramuto et al. 2016).
- *Phylogenomic cluster*: Based on the genome phylogeny clustering. Phylogenomic clusters were assigned to major branches that showed high bootstrap support values (>70%).
- *Epidemiological cluster*: Based on the time to the MRCA inferred from the reconstructed genome phylogeny. Epidemiological cluster members belonged to the same phylogenomic cluster and had a divergence date within a year prior to the start of our surveillance, that is, 2015 onwards.
- *Transmission cluster*: These were defined as viruses of the same epidemiological cluster which were independent introductions into the local population. As a conservative estimate, members of different transmission clusters diverged before the start of our local surveillance, that is, before January 2016.

The potential transmission networks within and between populations visiting the enrolled KHDSS health facilities were inferred in PopART package v1.7.2 using Templeton, Crandall and Sing (TCS) method with an epsilon of zero (Leigh and Bryant 2015). The networks were created for each identified phylogenomic cluster from the concatenated genome segments alignments.

### 2.11 Spatial analysis

We conducted a phylogeographic analysis to assess virus movement between the KHDSS locations and in relation to the rest of the world using methods implemented in BEAST v1.10.4 package. The analysis was implemented with a symmetric discrete trait approach and applied the Bayesian stochastic search variable selection (BSSVS) model (Lemey et al. 2009). To reduce complexity of the MCC, location states were categorised as ‘non-Kilifi’ or the specific health center regions. Phylogeographic inferences were visualised with the spatial phylogenetic reconstruction of evolutionary dynamics using data-driven documents (SPREAD3) v0.9.7.1c (Bielejec et al. 2016). To visualise the geographic migration of the virus over time, a D3 file was generated using SPREAD3 v0.9.7.1c. We used the KHDSS geo.json file. The resulting log files we used to calculate Bayes factor values for significant diffusion rates between discrete locations.

### 2.12 Statistical analysis

Numeric variable analyses were conducted using STATA v15.1 (StataCorp., College Station, TX, USA). Mean, median, and inter-quartile range were used to summarise continuous variables, whereas proportions were calculated for binary variables. Comparison between means and medians was done using *t*-test and median test, respectively. Patient distribution across the age groups and lineage distribution across demographic sub-categories were compared using the Wilcoxon Mann–Whitney *U* test and the independent *t*-test. Demographic and clinical characteristics among IBV positive patients sequenced versus those not sequenced and for the two lineages were compared using the Fisher’s exact test. Statistical significance (two-tailed) was set at *p*value of ≤0.05.

## 3. Results

### 3.1 Demographic characteristics

Between January and December 2016, a total of 5,647 NP swabs were collected from the nine KHDSS outpatient facilities and 574 NP/OP swabs from inpatients at KCH, Fig. 1b. Of these, 4.7 percent (267/5,647) from the outpatient facilities and 1.9 percent (11/574) of samples from KCH tested IBV positive by real-time RT-PCR. Sequencing attempt on 120/278 selected positives (43.2%, 109 from KHDSS outpatient facilities and 11 from KCH), yielded 111 whole genomes; 101 from KHDSS outpatient facilities and 10 from KCH, Fig. 1b. The demographic and clinical characteristics of the patients from whom genomes were obtained were similar to those for whom genomes were not obtained except for viral loads (as indicated by diagnostic RT-PCR *Ct* value) and health facility type (inpatient or outpatient), Table 1. The patient age among IBV positive patients ranged between 1 month and 85 years (mean: 9.6 years and median: 6 years). The proportion positive was highest in patients aged between 0 and 4 years (43.2%) followed by the 5–14 years age group (39.2%). Female patients accounted for most IBV positives (57.2%) and cough was the most common symptom in the IBV positive patients followed by fever and nasal discharge, Table 1.

### 3.2 Seasonality and representativeness of the sequenced samples

IBV were detected in all months of 2016 in the surveillance although the number of cases fluctuated from month-to-month, Fig. 1c. Notably each KHDSS health facility experienced a peak incidence at different months of the year but the majority fell between March and August. This coincided with one of the two rainy seasons in region. At the individual health facilities, some months had no IBV detections in the samples analysed. When all the enrolled health facilities were combined, sequence data were available from every month in 2016 except February and December. The fraction of samples from each health facility which were sequenced roughly reflected the total number of the positives that were detected in the specific health facility, Supplementary Fig. S2. At least one sample was sequenced from each of the enrolled health facilities and this enabled our interrogation of IBV transmission between the populations served by the enrolled health facilities.

**Figure 2.**
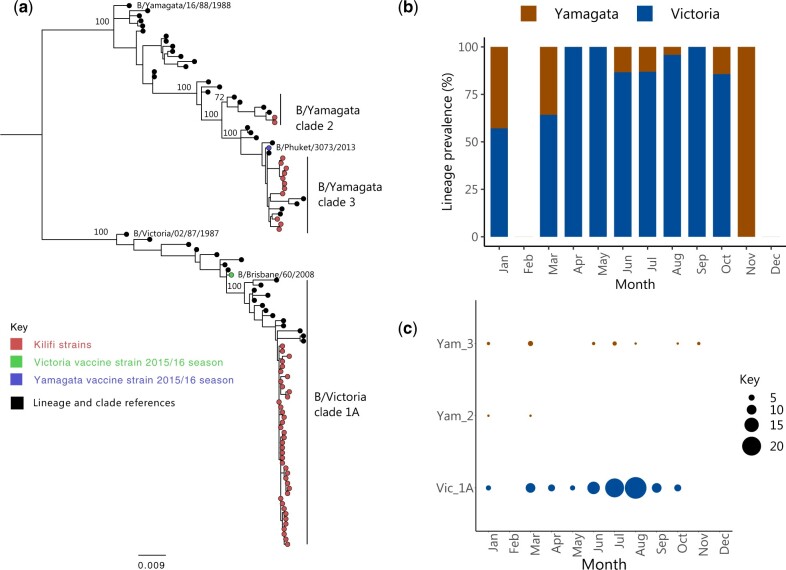
Identification of IBV lineages and clades that were in circulation in coastal Kenya in the 2016. (a) An ML phylogenetic tree based on the HA segment of samples we sequenced. From the surveillance, only unique HA sequences are included. The tree includes reference sequences of previously identified clades within B/Yamagata and B/Victoria lineages collected between 1987 and 2020. Kilifi sequences are shown with a red circle, the reference with a black filled circle. Strains included in the 2015–6 influenza vaccine are shown in a green filled circle for B/Victoria and blue filled circle for B/Yamagata. (b) The monthly prevalence of the B/Yamagata and B/Victoria lineage viruses across the 12 months the surveillance was undertaken. (c) The virus clades that were detected and their frequency across different months of the year 2016.

### 3.3 The B/Yamagata and B/Victoria lineages co-circulated in the study population

The HA phylogeny demonstrated that the two known IBV lineages were co-circulating: B/Victoria and B/Yamagata, Fig. 2a. Overall, the B/Victoria lineage predominated during this single year (84.7%) with all its sequenced viruses falling into clade 1A, Fig. 2b. The HA of B/Yamagata lineage viruses clustered within two genetically distinct known clades: clade 2 (*n* = 2) and clade 3 (*n* = 15). The B/Yamagata clade 3 viruses were detected in low numbers generally but throughout the year, whereas the B/Yamagata clade 2 viruses were detected only in January and March as shown in Fig. 2c. The demographic and clinical characteristics of patients infected by either B/Yamagata lineage or B/Victoria lineage was not statistically different, Supplementary Table S3.

**Table 3.** Divergence times and rates of nt substitution of gene segments of IBV.

	tMRCA			Substitution rate ×10^−3^		
Segment	Mean	Low 95% HPD	High 95% HPD	Mean	Low 95% HPD	High 95% HPD
PB2	Feb 1982	Nov 1977	Oct 1985	1.53	1.29	1.78
PB1	Jun 1979	Jan 1976	Oct 1982	1.33	1.16	1.51
PA	Nov 1979	Jul 1974	Sep 1984	1.46	1.28	1.66
HA	Dec 1980	May 1976	Jun 1985	1.97	1.70	2.24
PA	Nov 1979	Jul 1974	Sep 1984	1.46	1.28	1.66
NA	Mar 1983	Sep 1980	Aug 1985	1.92	1.67	2.21
MP	Mar 1983	Jun 1970	Apr 1987	1.44	1.26	1.69
NS	Jul 1968	Sep 1960	Jul 1975	1.24	1.00	1.48

### 3.4 Inter-lineage reassortment in the Kilifi IBV strains

Segment-specific phylogenies showed a clear separation into the B/Yamagata and B/Victoria lineages for the majority of the Kilifi viruses (*n* = 101, 90.1%) in all the segments: PB2, PB1, PA, HA, NA, NP, M, and NS (Supplementary Fig. S4). For the remainder viruses (*n* = 10), two inter-lineage reassortment events were suspected that were confirmed in GiRaF analysis, Supplementary Table S5. The first involving a single virus (B/Kilifi/114/2016/KCH/14-Oct-2016) that had B/Victoria lineage backbone including the HA segment but its PB2, PB1, PA, and MP segments clustered closely to B/Yamagata lineage viruses, Supplementary Fig. S4. Notably, for this virus, its NA and NP segments were distinct from the other B/Victoria lineage viruses. The second reassortment event involved nine B/Yamagata clade 3 viruses in which the NA and NP segments had been acquired from B/Victoria lineage but the backbone (i.e. all other segments) remained of B/Yamagata lineage. The B/Yamagata clade 2 viruses (*n* = 2) maintained a unique and unchanged constellation across all the eight segments.

### 3.5 Phylogenomic clusters in the Kilifi IBV 2016 epidemic

**Figure 3.**
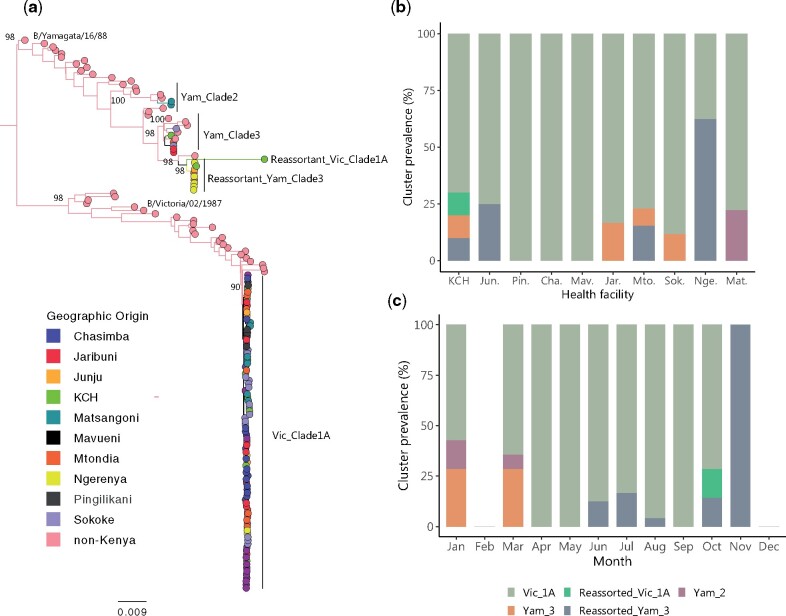
Genomic epidemiology of IBV in coastal Kenya. (a) An ML phylogenetic tree reconstructed from concatenated eight segments of IBV (*n* = 111) from the nine sampled KHDSS outpatient facilities and KCH. A total of fifty-one reference sequences (non-Kenya) were included. The taxa are indicated by filled circles at the tips and coloured by health facility of sampling. (b) The location of detection for the defined phylogenomic clusters. Their first three letters abbreviate names of the outpatient health facilities are. (c) The monthly prevalence of the defined phylogenomic clusters.

The ML phylogeny of the concatenated eight segments of the Kilifi IBV strains, including reference strains is shown in Fig. 3a. Multiple well-supported clusters were observed with evidence of mixing of virus samples from different health facilities. We assigned the five main branches including Kilifi strains with high bootstrap support (>70%) phylogenomic clusters, namely, (i), pure B/Victoria lineage clade 1A viruses (*n* = 93, 83.8%), (ii), reassortant B/Victoria lineage clade 1A virus (*n* = 1, 0.9%), (iii), pure B/Yamagata clade 2 viruses (*n* = 2, 1.8%), (iv), pure B/Yamagata clade 3 viruses (*n* = 6, 5.4%), and (v), B/Yamagata clade 3 reassortant viruses (*n* = 9, 8.1%). The KCH surveillance captured four of the five of these circulating phylogenomic clusters excepting only the pure B/Yamagata clade 2 viruses. Viruses of this clade were seen only in one KHDSS outpatient facility (Matsangoni, found furthest North in the KHDSS). Most other KHDSS outpatient facilities observed circulation of 1–2 clusters except Mtondia where three phylogenomic clusters were detected, Fig. 3b. Mtondia and Matsangoni are located along a Mombasa-Malindi highway, Fig. 1a. Of the 10 months that we obtained genome sequence data, in all except November, we detected the pure B/Victoria clade 1A viruses. The reassortant B/Yamagata clade 3 viruses were the second most persistent phylogenomic cluster with detection in 5 months of the 10 months, Fig. 3c. B/Yamagata clades 2 and 3 pure clusters were observed only at the beginning of the year, whereas reassorted B/Victoria clade 1A virus was detected only in October and only at the KCH facility.

### 3.6 Evolutionary dynamics of the Kilifi IBV strains

**Figure 4.**
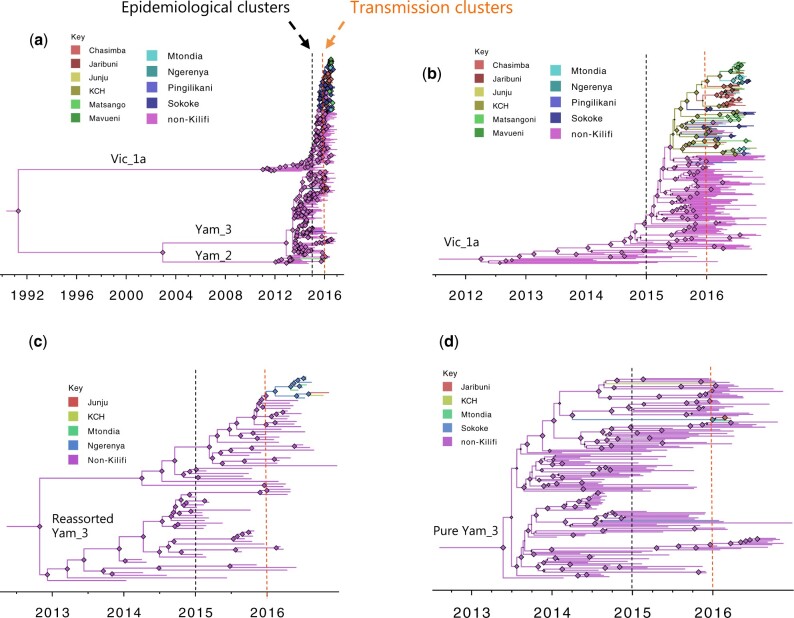
Time-resolved phylogenies of sub-samples of global genomes combined with Kilifi IBV genomes. The branches and the node shapes (size scaled by posterior support of the branch) are coloured by the most probable ancestral location inferred during BSSVS analysis. The threshold for epidemiological clusters (having the MRCA within the year preceding the start of our surveillance, between January and December 2015) is indicated by black dashed line, whereas the threshold of transmission clusters (i.e. occurred first as a single branch at the start of our surveillance in January 2016 with or without onward diversification) is indicated by the orange dashed line. (a) Shows combined non-reassortant strains. (b) Shows B/Victoria clade 1A viruses. (c) Shows reassortant B/Yamagata clade 3 viruses. (d) Shows pure B/Yamagata clade 3 viruses.

A strong linear relationship between root-to-tip genetic distance and sampling date was observed in all assessed phylogenomic clusters (*R*^2^ consistently >0.6), Supplementary Fig. S6. The Kilifi genomes fit well in the global continuum of observed diversity of the identified phylogenomic clusters. The time-resolved BEAST phylogenies for the combined non-reassortant viruses, and for main phylogenomic clusters in Kilifi (those with a sample size of > 2) are shown in Fig. 4. For the reassorted B/Yamagata clade 3 viruses, the global tMRCA was estimated to be around May 2013 (95% highest posterior density [HPD]: March 2013–August 2013), Fig. 4b and Table 2. The estimated nt substitution rates and tMRCA for the individual phylogenomic clusters are provided in Table 2. For all the individual phylogenomic clusters, tMRCA for the IBV viruses sampled in Kilifi during our surveillance occurred within 2015 except for the pure B/Yamagata clade 3 whose tMRCA occurred in October 2013 (95% HPD interval June 2012 to December 2014), Fig. 4d.

**Table 2.** The genomic evolutionary characteristics of the sequenced IBV strains in the surveillance stratified by phylogenomic cluster.

Phylogenomic cluster	No. of isolates (Kilifi)	tMRCA			Substitution rate × 10^−3^		
		Mean	Low 95% HPD	High 95% HPD	Mean	Low 95% HPD	High 95% HPD
Pure B/Vic/clade1A	209 (93)	Mar 2012	Jul 2011	Oct 2012	1.37	1.18	1.56
Reassorted B/Vic/clade1A	1 (1)	–	–	–	–	–	–
Pure B/Yam/clade2	46 (2)	Oct 2011	Nov 2010	Aug 2012	1.36	1.02	1.75
Pure B/Yam/clade3	157 (6)	May 2013	Mar 2013	Aug 2013	1.40	1.27	1.53
Reassorted B/Yam/clade3	79 (9)	Oct 2012	Mar 2012	April 2013	1.54	1.33	1.76

Yam for Yamagata, Vic for Victoria, substitution rate units are nts/site/year.

Using the global reference set collected from 1987 to 2020 and the Kilifi strains and the unique Kilifi strains, we estimated the tMRCA and nt substation rate for each of the segment, Table 3. The segments arrived at different tMRCA estimates for the included strains. The more recent tMRCA (March 1983) were from the NA and MP segments, whereas the earliest tMRCA (July 1968) was arrived at from analysis of NS segment. As expected, the highest nt substitution rates were observed with the HA segment (1.97 × 10^−3^) and NA segments (1.92 × 10^−3^), whereas the slowest substitution rate was observed with NS segment (1.24 × 10^−3^).

### 3.7 Global context of the 2016 Kilifi IBV strains

**Figure 5.**
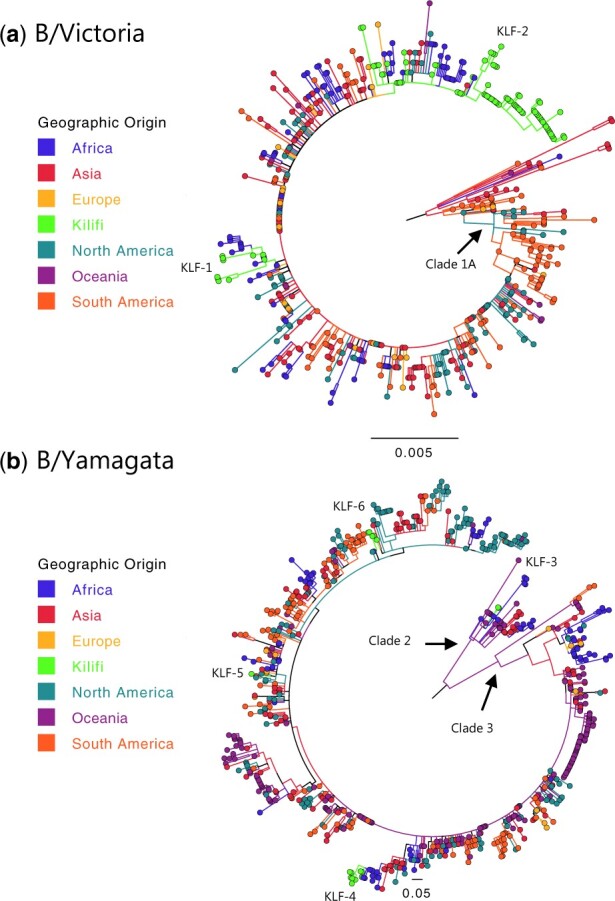
ML HA phylogeny of IBV strains sampled across the world between 2014 and 2016. (a) B/Victoria lineage (*n* = 481), (b) B/Yamagata lineage (*n* = 743). The taxa are shown as filled circles and are coloured differently for each geographic origin of the sample by continent. The newly sequenced Kilifi genomes are shown in a bright green colour and the major Kilifi clusters labelled (KLF-1–KLF-6).

We examined this using HA segment analysed by the ML approach. The global comparison dataset included 1,207 IBV strains sampled across six continents between 2014 and 2016. The phylogeny showed a clear bifurcation into two major clades corresponding to B/Victoria (*n* = 481) and B/Yamagata (*n* = 743) lineages (figure not shown). The ML phylogenies for the individual lineages are shown in Fig. 5. For both B/Victoria and B/Yamagata, the phylogenies confirmed that multiple distinct strains were in circulation in the Kilifi community in 2016 some of which had extensive local onward transmission, for example, B/Victoria clade 1A. The viruses clustering closest to the Kilifi IBV strains were commonly those detected in other African countries especially neighbouring Uganda and Tanzania (Supplementary Fig. S7).

By our set criteria (based on clustering with global sequences and divergence dates), we identified a total of seven epidemiological clusters and twenty-nine transmission clusters from the Kilifi IBV strains (see Figs 4 and 5**)**. The epidemiological cluster membership size (for Kilifi sequences) ranged from 1 to 93, whereas transmission cluster membership size varied from 1 to 28, Fig. 4. The vast majority of the Kilifi transmission clusters (23/29) were within the pure B/Victoria clade 1A.

### 3.8 Local phylogeography of the detected IBV strains

**Figure 6.**
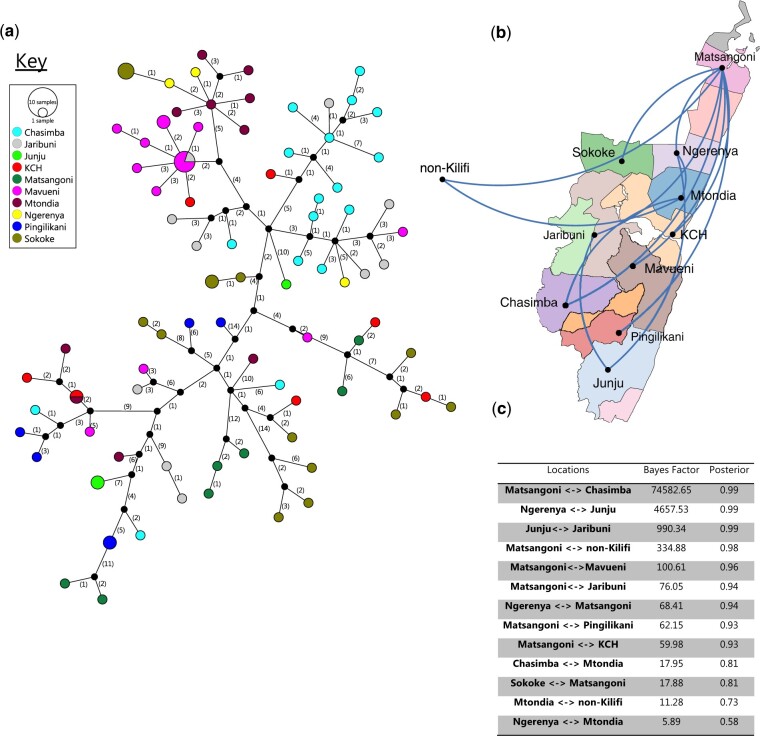
Possible transmission links between the Kilifi Victoria clade 1A viruses. (a) A TCS POPART network of the ninety-three newly sequenced pure Victoria clade 1A viruses. The vertices represent the concatenated genome haplotypes. The size of the vertex is proportional to the number of haplotypes (identical sequences) and is coloured by the health facility from which the sequenced sample was collected. The numbers shown on the edges represent the number of nt changes from one vertex (haplotype) to the next. (b) The phylogeography the newly sequenced pure Victoria clade 1A in comparison with non-Kilifi genomes (*n* = 116). The lines connecting the health facilities are shown only between location with support of a Bayes factor of >5. (c) The BF and posterior probability support for the links shown in (b).

The genetic relatedness Kilifi viruses within the same phylogenomic clusters by health facility is shown in Fig. 6 (for B/Victoria 1A) and Supplementary Fig. S8 (for both B/Yamagata pure and reassorted clade 3). In some of the KHDSS facilities, it was clear that a dominant transmission cluster existed, for example, some of the pure Victoria 1A variants for Chasimba, Mavueni, and Mtondia, whereas others had no clear dominant transmission cluster, Fig. 6a. Furthermore, the phylogeographic analysis showed that IBV Victoria IA viruses were commonly getting into and out of the KHDSS area through the Matsangoni area, Fig. 6b and c. The Matsangoni health facility had connection with seven of the nine other health facilities. Although the Yamagata clade 3 clusters had KCH as their link to the rest of the world, the sample size was relatively small and KCH is a referral facility thus a direct link cannot be concluded, Supplementary Fig. S8.

## 4. Discussion

Detailed phylodynamic and transmission studies on influenza in low-income settings in the tropics remain sparse. This is despite these regions bearing a disproportionately large influenza burden (Katz et al. 2012; Byarugaba et al. 2016). Here, through a comprehensive genomic analysis, we show that the year-round circulation of IBV in a coastal region of Kenya (covering ∼900 km^2^) in 2016 (Nyiro et al. 2018) was occasioned by co-circulating IBV clades and viral clusters within both B/Yamagata and B/Victoria lineages. Furthermore, we show that that the epidemic season was instigated by up to twenty-nine independent strain introductions, some of which were inter-lineage reassortants. The genomic analysis recognised extensive local spread of the new IBV strains once introduced accompanied with significant accumulation of nt substitutions.

The B/Victoria lineage predominated the IBV season we observed, being responsible for > 80 per cent of the IBV infections we sequenced. These finding are congruent with a recent IBV report from Kenya of a surveillance study across ten sites from 2012 to 2016 (Emukule et al. 2019). The authors observed that in the 2016 season, IBV was the predominant influenza type in the enrolled health facilities (just like at this coastal Kenya site, Nyiro et al. 2018) and the B/Victoria lineage was the predominant IBV lineage. Here through our detailed genomic analysis, we extend these earlier observations to show that this season was predominated by B/Victoria clade 1A strains and the co-circulating B/Yamagata viruses were of clades 2 and 3, and there were at least two inter-lineage reassortant strains in circulation.

Currently available seasonal influenza vaccines require periodic update to better match circulating influenza strains (Hampson et al. 2017). Both trivalent influenza vaccines that have a representative H3N2, H1N1, and IBV strain (of either B/Victoria or B/Yamagata lineage) and quadrivalent vaccines that have a representative H3N2, H1N1, and both IBV lineages (Victoria and B/Yamagata) are available (Grohskopf et al. 2019). Currently, Kenya does not have a national influenza vaccination policy (Dawa et al. 2019). In this study, although B/Victoria lineage was predominant, our findings support the notion of deployment of quadrivalent influenza vaccines for optimal vaccine effectiveness. The impact of the additional diversity we observed within B/Yamagata lineage (2 antigenically distinct clades) and the emergence of reassortant viruses on the overall vaccine effectiveness require further investigation.

IBV inter-lineage reassortment is well recognised in literatures (Dudas et al. 2015; Tewawong et al. 2017; Monamele et al. 2018). For instance, reassortant B/Yamagata lineage viruses with a B/Victoria NA were recently reported in Cameroon (2014–7; Monamele et al. 2018). Here, we identified two inter-lineage reassortment events: 1, B/Yamagata lineage viruses that had acquired NP and NA segments from B/Victoria lineage viruses and 2, a B/Victoria lineage virus that had acquired PB2, PB1, PA, and MP from B/Yamagata clade 3 viruses. Previous studies observed that IBV reassortant viruses tend to circulate at a low prevalence and do not persist over epidemics (Chi et al. 2005; Chen et al. 2007). In the current study, the reassorted B/Yamagata clade 3 appeared to transmit for at least 6 months, whereas the reassortant B/Victoria clade 1A strain had single time point detection. Notably, the latter reassortant was unusual given the co-segregation of PB2, PB1, and PA. Previous analysis noted that PB2, PB1, and HA segments tend to segregate together due to more compatibility (Dudas et al. 2015). A follow-up study to investigate the fate, clinical, and epidemiological impact of the reassortant strains we observed here will be useful.

Some studies have associated B/Victoria lineage infections with more severe disease compared with B/Yamagata lineage infections, whereas other did not find such relationship (Caini et al. 2018b; Emukule et al. 2019). In the current study, we did not observe a significant difference in lineage distribution between inpatients and outpatients. Furthermore, the genome phylogenies observed interspersing of strains that were found in the inpatients and those found in mild ARI outpatient cases. These observations suggest that it is host rather than viral factors that are most critical in determining IBV disease severity. Furthermore we found that IBV infections were most frequent among 0–14 year olds, and lineage distribution did not appear to be influenced by age unlike what has been reported in some previous studies (Horthongkham et al. 2016; Korsun et al. 2017; Yang et al. 2018).

The inclusion of regional and global genomes deposited in GISAID significantly improved the power of our phylodynamic analyses and showed that the Kilifi IBV diversity was part of the global continuum. For example, we determined that the reassortant Yamagata clade 3 viruses were circulating in several other countries including Uganda, Tanzania, Rwanda, Congo, Nigeria, Cote D’Ivoire, Mali, Burkina Faso, Indonesia, Laos, Bangladesh, Nepal, Singapore, Japan, and USA. By tMRCA analysis, we found that the reassortment event that resulted in this cluster occurred around October 2012 (95% HDP March 2012 to April 2013). However, we did not find a close relative in the database to the reassortant Victoria clade 1A. Our repeated bioinformatics analysis of the raw short-read data of this sample reproduced the reassorted genome.

The phylogeographic analyses demonstrated IBV migration both into and out of the KHDSS area. The Kilifi IBV genomes seemed to frequently have their close relatives in neighbouring Uganda and Tanzania. This observation is one that requires a follow-up investigation to test the hypothesis that new influenza epidemics are likely to be seeded from neighbouring East African countries than distant countries. Within the KHDSS area, for the phylogenomic cluster that had significant sample size, virus seeding seemed to start from Matsangoni. The area to the north of the KHDSS has two key touristic towns (Watamu and Malindi) and their proximity to Matsangoni might explain the virus entry via Matsangoni. Further investigation is required to confirm this hypothesis.

This study had some limitations. First, the sequencing was undertaken for only a single year period. Thus we cannot conclude on the long-term consistency of the observed IBV transmission patterns and the fate of the identified reassortant strains. Second, we sequenced only a fraction of the identified IBV positives (∼40%). The prioritised samples were selected on the basis of anticipated probability of successful sequencing inferred from the sample’s viral load as indicated by the diagnostic *Ct* value. Such a strategy ultimately avoided sequencing some samples that may have been critical in reconstructing the true transmission networks and may bias cluster prevalence. However, the demographic and clinical characteristics of the sequenced and not sequenced patients were similar except for their viral load. Third, the KHDSS outpatient facilities surveillance collected a maximum of fifteen samples/week/site. This non-exhaustive sampling at the facilities may have introduced bias in the inferred lineage/clade prevalence and transmission networks.

In conclusion, our genomic analysis of IBV confirms that B/Victoria (clade 1A) and B/Yamagata (clades 2 and 3) lineage viruses were in co-circulation together with two inter-lineage reassortant variants in coastal Kenya in 2016. The co-circulation of divergent IBV viruses complicates the optimal selection of influenza vaccine strain components for local use. As Kenya formulates its influenza vaccination policy, the choice of broad immunity (Epstein 2018) or more valence vaccines (e.g. quadrivalent regimen, Dbaibo et al. 2019) should be considered. Furthermore, this study demonstrates the benefits of analysis of full-length IBV genomes. In addition to providing a clearer understanding of locally circulating viral diversity, a high-resolution tracking of transmission of IBV strains was achievable at a scale impossible with single or few segment analysis. That in a single season up to twenty-nine independent IBV introductions occurred demonstrates the challenge of controlling local influenza epidemics by targeted approaches, for example, sub-population vaccination, patient quarantine, or institutional closures as previously observed (Holmes et al. 2011). Future studies should combine genomic data with various epidemiological data (e.g. host migration, immunity profiles, population densities, social contact patterns) to elucidate patterns of IBV infection and spread in this setting for better-informed control strategies.

### Data availability

All data generated or analysed during this study has been deposited to the VEC Data Repository in Havard Dataverse under the doi: https://doi.org/10.7910/DVN/OE6TS2. The sequence data are deposited on the GISAID database under the accession numbers: EPI_ISL_336258 and EPI_ISL_336282–EPI_ISL_336395.

## Acknowledegments

We thank the study participants for providing samples, the field workers for collecting epidemiological data and the clinical samples, and members of the Virus Epidemiology and Control Research Group at KEMRI—Wellcome Trust Research Programme for laboratory processing of the samples and stimulating discussions. We thank Dr John Barnes and Dr David E. Wentworth of the Centers for Disease Control and Prevention, Atlanta, USA for their input in setting up the MiSeq whole-genome sequencing assays in Kenya. We acknowledge the authors, originating and submitting laboratories of the sequences from GISAID™ EpiFlu database on which this research is based, Supplementary Table S9. All submitters of data may be contacted directly via the GISAID website www.gisaid.org.

## Funding

This study was funded by the Wellcome Trust (102975, 203077). The authors F.M.N., D.C.O and C.N.A. were supported by the Initiative to Develop African Research Leaders (IDeAL) through the DELTAS Africa Initiative (DEL-15-003). The DELTAS Africa Initiative is an independent funding scheme of the African Academy of Sciences (AAS)’s Alliance for Accelerating Excellence in Science in Africa (AESA) and supported by the New Partnership for Africa’s Development Planning and Coordinating Agency (NEPAD Agency) with funding from the Wellcome Trust (107769/Z/10/Z) and the UK government. The views expressed in this publication are those of the authors and not necessarily those of AAS, NEPAD Agency, Wellcome Trust or the UK government. This paper is published with the permission of the Director of KEMRI. 

**Conflict of interest:** None declared. 

## Supplementary Material

veaa045_Supplementary_Data
