# Supplementary material for: Epidemiological and evolutionary dynamics of influenza B virus in coastal Kenya as revealed by genomic analysis of strains sampled over a single season
Source: Virus Evol. 2020 Aug 16;6(2):veaa045. doi: 10.1093/ve/veaa045 (PMC7959010; doi:10.1093/ve/veaa045)
Supplement: veaa045_Supplementary_Data [file ve_6_2_veaa045_s7.zip › supplementary_Material_01Jun2020.docx]

**Supplementary Material**

**Epidemiological and evolutionary dynamics of influenza B virus in coastal Kenya as revealed by genomic analysis of strains sampled over a single season**

Festus M. Nyasimi^1,2, #^, D. Collins Owuor^1^, Joyce M. Ngoi^1^, Alexander G. Mwihuri^1^, Grieven P. Otieno^1^, James R. Otieno^1^, George Githinji^1^, Joyce U. Nyiro^1^, D. James Nokes^1,2,3^ and Charles N. Agoti^1,2^

**Author Affiliations**

^1^Epidemiology and Demography Department, Kenya Medical Research Institute (KEMRI) –Wellcome Trust Research Programme, Kilifi, Kenya. ^2^Department of Public Health, School of Human and Health Sciences, Pwani University, Kilifi, Kenya. ^3^School of Life Sciences and Zeeman Institute of Systems Biology and Infectious Disease Epidemiology Research (SBIDER), University of Warwick, UK.

**S1 Table.** Global IBV genomic datasets used to place the Kilifi genomes on the global context.

| Continent | Total Available ^€^ | Global context sub-sample^$^ | 2^nd^ Phylogenomic cluster sub-sample^¥^ |
| --- | --- | --- | --- |
| Africa | 184 | 184 | 78 |
| North America | 2231 | 250 | 73 |
| South America | 240 | 240 | 53 |
| Oceania | 230 | 230 | 57 |
| Europe | 53 | 53 | 13 |
| Asia | 873 | 250 | 75 |
| Total | 3811 | 1207 | 349 |

^€^ Search conducted in GISAID database on 15-Jan-2020. Only IBV genomes with collection dates between January 2014 and December 2016 were eligible for inclusion.

^$^Used in the maximum likelihood phylogenetic reconstruction.

^¥^ Used in evolutionary analysis in BEAST program to estimate phylogenomic cluster genomic substitution rates and dates divergence date of tMRCA.

**Note:** A third sub-sample was selected across the period 1987-2020 and this was used to assign lineage and clades to the Kilifi IBV strains and in segment-by-segment evolutionary analyses.

**S2 Figure.** Distribution of the sequenced samples. Panel (a) shows the number of IBV positives across all months in 2016 stratified by the sequencing status. Panel (b) presents the number of IBV positives by month and health facility that we genome sequenced. The size of the circle is proportional to the number of samples (smallest representing one and largest representing 8 samples).

**S3 Table.** Comparison of the clinical and demographic characteristics the patients who were infected by Yamagata and Victoria lineages

| Characteristics | Victoria (n=94) | Yamagata (n=17) | p value |  |
| --- | --- | --- | --- | --- |
| Health facility |  |  |  |  |
| Inpatient (KCH) | 8 | 2 | 0.694 |  |
| Outpatient (KHDSS) | 86 | 15 |  |  |
| Age (years) |  |  |  |  |
| Mean (SD) ^€^ | 9.4 (14.3) | 11.5 (19.9) | 0.588 |  |
| Median (IQR) | 6 (2-11) | 7 (2-10) | 0.655 | |
| Age class (Years) |  |  | 0.680 |  |
| 0-4 | 45.8% | 35.3% |  |  |
| 5-14 | 40.4% | 52.9% |  |  |
| 15-34 | 8.5% | 5.9% |  |  |
| 35-64 | 3.2% | 0 |  |  |
| ≥ 65 | 2.1% | 5.9% |  |  |
| Gender |  |  |  |  |
| Female | 57.4% | 64.7% | 1.000 |  |
| Male | 42.6% | 35.3% |  |  |
| Symptoms | n=86 | n=15 |  |  |
| Fever* | 79.1% | 80.0% | 1.000 |  |
| Cough* | 96.5% | 100% | 1.000 |  |
| Nasal discharge* | 79.1% | 73.3% | 0.735 |  |
| Breathing difficulty* | 8.1% | 0.0% | 0.590 |  |
| Viral load |  |  |  |  |
| Mean (SD) ^€^ | 25.0 (2.3) | 24.8 (2.2) | 0.754 |  |
| Median | 25.0 (23.5-26.5) | 24.4 (24.4-26.4) | 0.570 |  |
| * Symptoms proportions were calculated for outpatients only as all inpatients have severe symptoms. | | | |  |

^€^SD stands for Standard of Deviation

**S4 Figure.** Segment specific ML phylogenetic trees for each of the eight IBV genomic segments reconstructed from the 111 strains we genome sequenced in the current study. The filled circle tips represent the taxa which are colored as per the lineage identified from the HA phylogeny; teal blue for Victoria lineage and Maroon for Yamagata lineage. The position of the reassorted Victoria lineage that had acquired Yamagata lineage PB1, PB2 PA and MP segments is indicated by red and magenta arrows. The Yamagata reassortant cluster that had acquired Victoria lineage NA and NP segments has a red star next to the branch node.

**S5 Table.** A GiRAF analysis summary table showing the segment-segment report for individual segment reassortant candidates in brackets and the confidence level for each candidate taxa.

| Segment | PB2 | PB1 | PA | HA | NP | NA | MP | NS |
| --- | --- | --- | --- | --- | --- | --- | --- | --- |
| PB2 | - |  |  |  |  |  |  |  |
| PB1 | 1.000 (114) | - |  |  |  |  |  |  |
| PA | 0.999 (103)  0.999 (114) 0.996 (008,022) | none | - |  |  |  |  |  |
| HA | none | 1.000 (114) | 0.999 (103)  0.999 (114) | - |  |  |  |  |
| NP | none | none | 1.000 (114) | None | - |  |  |  |
| NA | none | 1.000 (114) | none | 0.999 (A)  0.999 (B) | none | - |  |  |
| MP | 0.999 (008,022) | 1.000 (114) | 0.999 (114) | none | 0.999 (C) | none | - |  |
| NS | none | 1.000 (114) | 1.000 (114) | none | 0.999 (A) | 0.999 (A) | 0.991 (114) | - |

A: 055,070,071,085,091,096,97,103,108

B: 010,016,022,023,025,026,055,070,071,085,091,096,097,103,107,108,114

C: 10,16,23,25,26,10

**S6 Figure**. Temporal phylogenetic signal in the assigned phylogenomic clusters. This was evaluated by root-to-tip regression of genetic distances derived from reconstructed ML genome phylogenies of combined Kilifi and global genomes assigned to the respective phylogenomic clusters. Data points are coloured by health facility and are shown in black for sequences from outside Kilifi. The regression equation, R^2^ and standard error 95% CI region are shown on the individual plots. Presence of a temporal signal in genetic distances was evident for all phylogenomic clusters.

**S7 Figure**. This figure is on a separate file

**S8 Figure.** Possible transmission links between the Kilifi Yamagata clade 3 viruses. Top panel shows minimum spanning POPART networks of the sequenced Yamagata clade 3 viruses by cluster. The vertices represent the concatenated genome haplotypes. The size of the vertex is proportional to the number of haplotypes (identical sequences) and is colored by the health facility from which the sequenced sample was collected. The numbers shown on the edges represent the number of nt changes from one vertex (haplotype) to the next. Middle panel shows the phylogeography the Kilifi Yamagata clade 3 phylogenomic clusters in comparison with non-Kilifi genomes. The lines connecting the health facilities are shown only between location with support of a Bayes Factor of > 5. Bottom panel shows the Bayes Factor and posterior probability support for the links shown in middle panel.
