# Supplementary material for: Epidemiological and evolutionary dynamics of influenza B virus in coastal Kenya as revealed by genomic analysis of strains sampled over a single season
Source: Virus Evol. 2020 Aug 16;6(2):veaa045. doi: 10.1093/ve/veaa045 (PMC7959010; doi:10.1093/ve/veaa045)

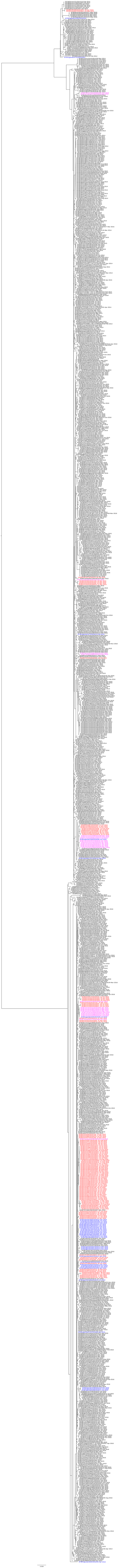

Supplement: veaa045_Supplementary_Data [file S7 Figure.pdf › S7 Figure.pdf]
